# Label-Free Imaging of Blood Vessels in Human Normal Breast and Breast Tumor Tissue Using Multiphoton Microscopy

**DOI:** 10.1155/2019/5192875

**Published:** 2019-06-25

**Authors:** Gangqin Xi, Ning Cao, Wenhui Guo, Deyong Kang, Zhong Chen, Jiajia He, Wenjiao Ren, Tingfeng Shen, Chuan Wang, Jianxin Chen

**Affiliations:** ^1^Key Laboratory of Optoelectronic Science and Technology for Medicine of Ministry of Education, Fujian Provincial Key Laboratory of Photonics Technology, Fujian Normal University, Fuzhou 350007, China; ^2^Department of Plastic Surgery, Zhangzhou Affiliated Hospital of Fujian Medical University, Zhangzhou, 363000 Fujian, China; ^3^Department of Breast Surgery, Fujian Medical University Union Hospital, Fuzhou 350001, China; ^4^Department of Pathology, Fujian Medical University Union Hospital, Fuzhou 350001, China

## Abstract

Blood vessels are the important components of the circulatory systems that transport blood throughout the human body and maintain the homeostasis of physiological tissues. Pathologically, blood vessels are often affected by diseases, leading to the formation of unstable, irregular, and hyperpermeable blood vessels. In the tumor microenvironment, abnormal leakage of tumor blood vessels is related to the histological grade and malignant potential of tumors and may also facilitate metastasis of cancer. Visual diagnosis of blood vessels is very important for us to understand the occurrence and development of diseases. Multiphoton microscopy (MPM) is a potential label-free diagnostic tool based on second harmonic generation (SHG) and two-photon excited fluorescence (TPEF). MPM can effectively observe the morphological changes of biological tissues at the molecular and cellular levels. In this work, we demonstrate that label-free MPM can be used to visualize the microstructure of blood vessels in human normal breast and breast tumor tissue. Moreover, MPM can monitor the changes of blood vessels in tumor microenvironment. These results show that the MPM will become a promising technique for clinicians to study the properties of the microstructure of the blood vessels.

## 1. Introduction

Blood vessels are the primary components of the circulatory system. According to their structure and function, blood vessels are classified as arteries, capillaries, and veins [1]. The importance of vascular analysis has been supported by the introduction of new medical techniques aimed at enhancing vascular visualization in clinical practice. Blood vessel imaging plays an important role in different clinical fields, such as brain [2], tumor [3], ophthalmology [4], and neurosurgery [5], whether in diagnosis, treatment planning, and implementation or in evaluation and follow-up of treatment results. Vascular imaging can measure vascular permeability and analyze the abnormalities of cells on the wall of blood vessels. They can pinpoint the location of angiogenesis, assess vascular heterogeneity, and distinguish the characteristics of angiogenesis and normal blood vessels [6]. In traditional histopathology, hematoxylin and eosin (H&E) staining is a gold standard for pathological diagnosis. However, the H&E staining procedure requires a lot of time, including formalin fixation and paraffin embedding. Therefore, it cannot be used for real-time imaging. Computed tomography (CT), magnetic resonance imaging (MRI), and positron emission tomography (PET) provide noninvasive images of blood vessels in animals and humans. CT allows 3D visualization of the vessels and its surrounding structures. In spite of excellent image quality, CT uses ionizing radiation and exogenous contrast agents, which are harmful. MRI works based on the contrast related to the differences in the density of the proton. It does not use ionizing radiation but requires contrast agents. However, it also has some disadvantages: long scan time, probable need for strong magnetic fields for better signal-to-noise ratio, and being not ideal for real-time imaging. PET has resolutions of a few millimeters and can only detect lesions in larger vessels. Although MRI, CT, and PET imaging can be used to monitor abnormal blood vessels in tumors, they have some limitations [7–9]. Therefore, it is necessary to develop noninvasive and label-free methods to better identify blood vessels.

In recent years, MPM has become a key technology for biological tissues imaging at the cellular level and can provide high-contrast structural images in the depth of tissues [10–12]. Both types of nonlinear interactions occur in biological tissues without the use of exogenous stains. In this context, we use MPM to visualize the vascular structure of human breast tissue. MPM clearly reveals the layered structure of the blood vessel wall and can clearly identify normal blood vessels and tumor-related blood vessels.

## 2. Materials and Methods

### 2.1. Sample Preparation

Informed consent for this research was obtained from all patients, and the Institutional Review Board of the Affiliated Union Hospital of Fujian Medical University approved this study. A total of 25 breast cancer patients participated in this study. Normal mammary tissues were taken 6 cm away from the tumor margin. They were processed with a part of standard pathologic procedures, including formalin fixation, alcohol dehydration, and paraffin embedding. Every paraffin block sample was cut into three 5 *μ*m thick serial tissue slices by Ultra-Thin Semiautomatic Microtome in the pathology laboratory. Before imaging, the samples were deparaffinized by alcohol and xylene. The middle section was used to stain with hematoxylin and eosin (H&E) and make the histological examination with standard light microscopy. The remaining two adjacent sections were for MPM imaging.

### 2.2. Multiphoton Microscopic Imaging System

The MPM imaging system is a commercial device combined with a Zeiss LSM 880 laser scanning microscope and a mode-locked Ti : sapphire laser, tunable from 690 nm to 1064 nm. In our experiments, the excitation light of 810 nm was chosen for MPM imaging. In channel mode, images can be collected in two separate channels simultaneously. One channel of detecting SHG signals (green color coded) was set from 395 nm to 415 nm, whereas the other channel of detecting TPEF signals (red color coded) was set from 428 nm to 695 nm. In lambda mode, spectral images were obtained by collecting emission signals between 389 and 716 nm. In order to obtain clear large-area images, we chose a 20x (Plan-Apochromat, NA = 0.8, Zeiss, Jena, Germany) objective in this work for channel mode and chose a 63x (Plan-Apochromat, NA = 1.4, Zeiss, Jena, Germany) oil immersion objective for lambda mode.

## 3. Results

### 3.1. Identifying the Structure of the Blood Vessel Wall via MPM

To elucidate whether MPM has the potential to identify the structure of the vascular wall, we performed MPM images on the artery wall of unstained human breast tissue sections. Except for the capillaries, the wall of blood vessels from the lumen surface to the outside is tunica intima, tunica media, and tunica adventitia [13]. Collagen and elastin are important components of the vascular wall that contribute to the strength of the vessels wall, and the quantity and structure of the collagen are primarily what define the mechanical properties of the vessel wall [14].

**Figure 1:**
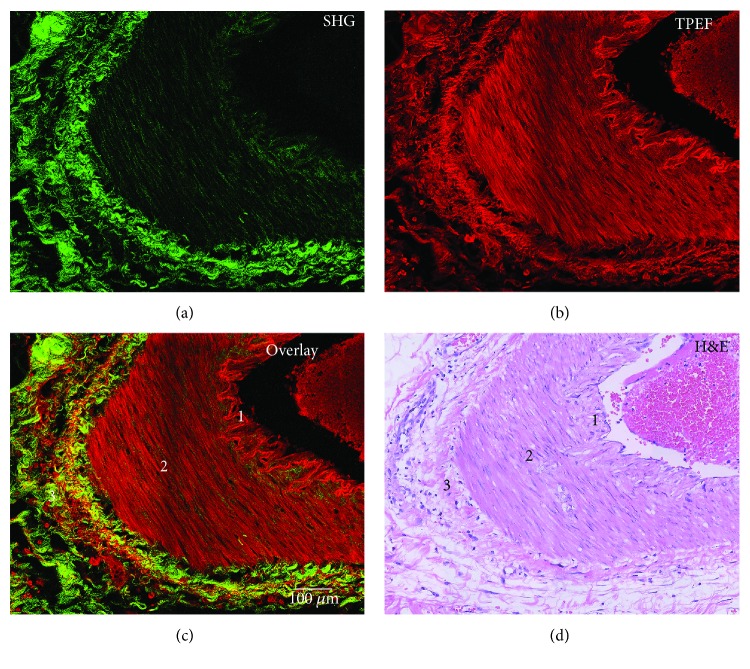
MPM images and the corresponding H&E-stained image of the wall of blood vessel: (a) SHG image (green color coded), (b) TPEF image (red color coded), (c) overlaid SHG/TPEF image, and (d) H&E-stained image. Position 1: tunica intima; Position 2: tunica media; Position 3: tunica adventitia. Scale bar: 100 *μ*m.

Figures 1(a) and 1(b) are SHG and TPEF images, respectively. In Figure 1(c), there are obvious differences in the layers of the vascular wall and the interface between the layers because of the different endogenous signals of the layers. Hence, the position of the three layers of the blood vessel wall was easily discernible. In the MPM images, the intima (Position 1) produces a strong TPEF signal (Figure 1(b)) and a weak SHG signal (Figure 1(a)). The intima consists of the endothelial cells and subendothelial layer (containing a small amount of collagen fibers and elastic fibers). The image displays a strong TPEF signal from endothelial cells and elastic fibers while a weak SHG signal from collagen. On the transverse sections of a vessel, the intima appears as a highly wavy structure because the wall of the vessel is constricted [13]. The intima of vessels was readily discerned by MPM, compared with H&E-stained image in Figure 1(d). The tunica media (Position 2) is composed of mostly smooth muscle cells and few elastin and collagen fibers. The MPM images of the tunica media display a strong TPEF signal from smooth muscle cells and elastic fibers (Figure 1(b)) while a weak SHG signal from collagen (Figure 1(a)). The tunica adventitia contains elastin fibers and dense collagen fibers [13]. In the adventitia (Position 3), the images display a strong SHG signal from collagen fibers (Figure 1(a)) and a TPEF signal from elastin fibers (Figure 1(b)). Overlaying image shows structural details of the blood vessel wall with high contrast. Moreover, compared with the H&E-stained image, the components of the blood vessel wall can be seen more clearly via the MPM images.

### 3.2. Spectral Analysis of the Layer Structure of the Blood Vessel Wall

**Figure 2:**
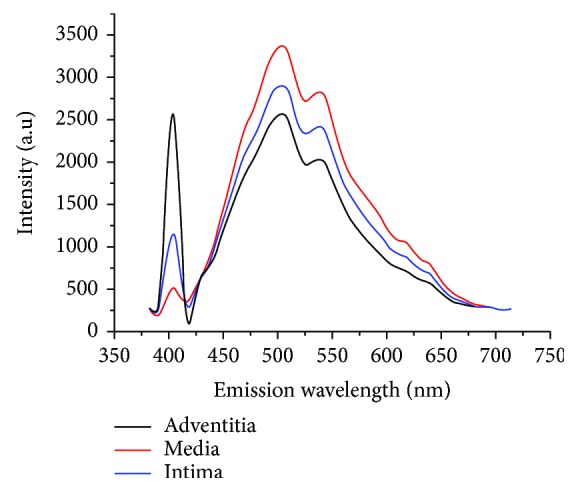
The multiphoton emission spectra of intima (blue), media (red), and adventitia (black) from the blood vessel wall.

Because the endogenous components of the vascular wall are different, spectral imaging of the vascular wall was also carried out to elucidate the origin of the nonlinear optical signals. The tissue sections were excited at 810 nm excitation wavelength for collecting emission spectra, and nonlinear emission spectra were collected between 389 and 716 nm using spectral detector under the lambda mode.  Figure 2 shows the emission spectra from intima (blue), media (red), and adventitia (black) of the blood vessel wall. The emission spectra of the blood vessel wall are composed of two parts: a narrow emission peak about half of the excitation wavelength (810 nm) of SHG signal and a broad emission spectrum of TPEF signal from 430 and 716 nm. Comparing the SHG and TPEF signal intensity of the three layers of the vascular wall, it was found that the SHG signal of intima was stronger than that of media, while the TPEF signal of intima was weaker than that of media. The tunica adventitia exhibits a strong SHG signal because it contained dense collagen fibers.

### 3.3. Identifying Various Blood Vessels in Normal Breast Tissues via MPM

**Figure 3:**
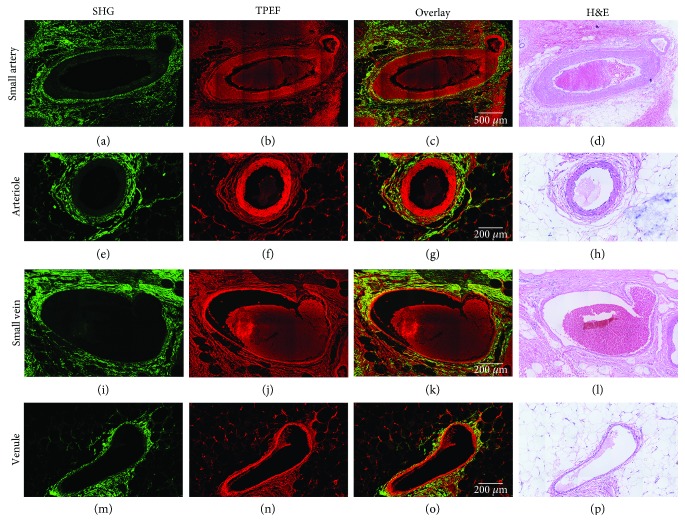
SHG, TPEF, overlaid SHG/TPEF, and corresponding H&E-stained image of the small artery (a–d), the arteriole (e–h), the small vein (i–l), and the venule (m–p). Scale bars: 500 *μ*m (first row) and 200 *μ*m (others).

To demonstrate the ability of MPM to detect the architecture of different blood vessels, we also imaged various vessels. In this paper, we identified four main types of blood vessels according to the structures of the wall and the diameter of the cavity.  Figure 3 shows the MPM images and the corresponding H&E-stained image of different types of blood vessels. The first row in Figure 3 displays the MPM images (a, b, and c) and the corresponding H&E-stained image (d) of a small artery, as can be seen, the three layers of the vessel wall are captured with clarity by MPM. There are a large number of smooth muscles in the walls of small arteries, which can contract or relax to change the amount of blood delivered [13, 15]. The second row in Figures 3(e)–3(h) depicts the structure of the arteriole; it has a lumen less than 300 *μ*m in diameter. Arterioles control blood flow in capillary beds by contracting or expanding the lumen, so the vascular wall contains several concentric rings of smooth muscle [13]. The relatively obvious three-layer structure of arteriole can also be seen from the MPM images, which is consistent with H&E-stained image. Figures 3(i)–3(l) display the SHG, TPEF, overlaid SHG/TPEF, and corresponding H&E-stained image of the small vein, respectively. Small veins have much larger lumen and thinner walls than small arteries, which is associated with less blood pressure in the veins than in the arteries. The veins still have the three basic layers (intima, media, and adventitia). The walls of the small veins consist of intima with endothelium, a thin media with a few smooth muscle cells and elastic fibers, and a very thin tunica externa consisting of connective tissue [13]. Figures 3(m)–3(p) show the MPM and H&E-stained images of the venule. It is a very small blood vessel in the microcirculation. Venules range from about 50 *μ*m to 200 *μ*m in diameter. Compared with small veins, venules have smaller lumen diameter, and the three-layer structure of the vascular wall is also obvious.

### 3.4. MPM Imaging of the Abnormal Blood Vessels in Tumor Microenvironment

**Figure 4:**
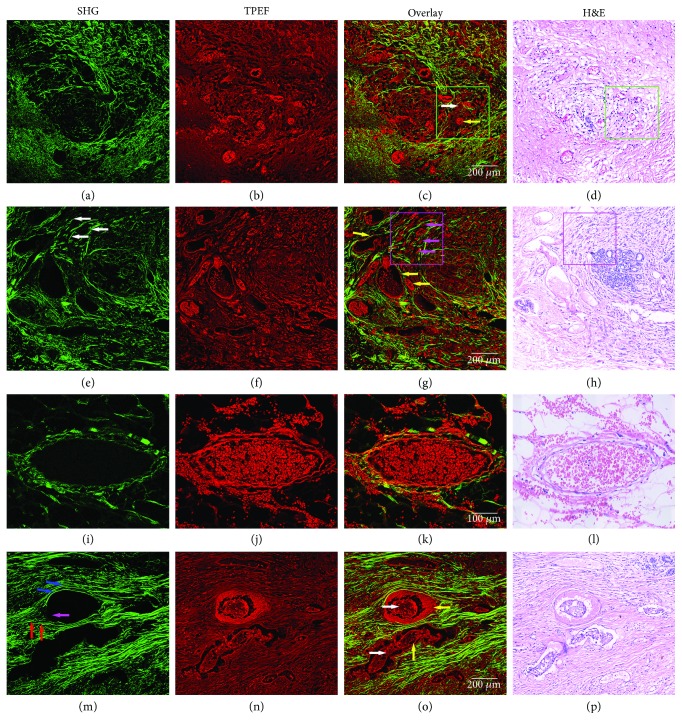
SHG, TPEF, overlaid SHG/TPEF and corresponding H&E-stained image of the abnormal blood vessels in tumor microenvironment: (a–d) the macrophages and numerous microvessels around them (macrophages of white arrow and microvessels of yellow arrow), (e–h) the irregular blood vessels (blood vessels of yellow arrow, tumor cells of pink arrow, collagen fibers of white arrow), (i–l) blood vessels with abnormal leakage, and (m–p) blood vessels invaded by tumor cells (blood vessels of yellow arrow, tumor cells of white arrow, basement membrane of pink arrow, collagen fibers of blue and red). Scale bars: 100 *μ*m (third row) and 200 *μ*m (others).

**Figure 5:**
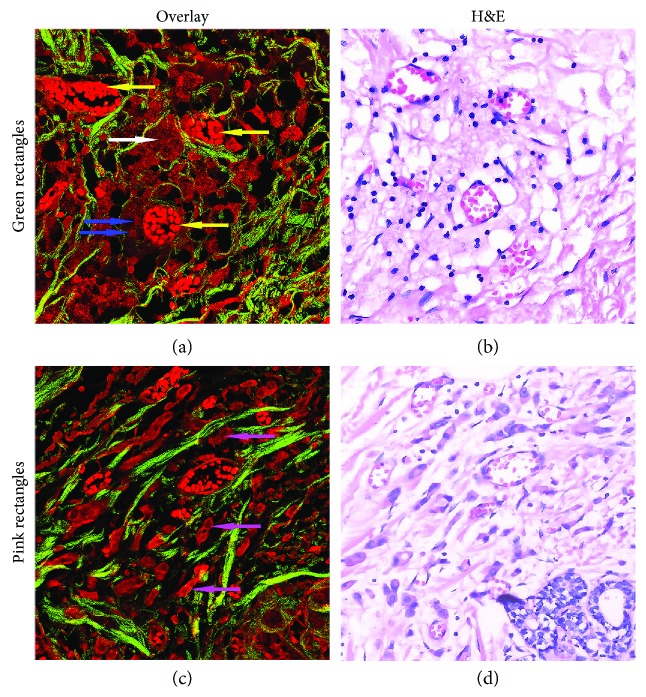
The enlarged views of the green rectangles in Figures 4(c)–4(d) and the pink rectangles in Figures 4(g)–4(h).

Tumor vessels exhibit many structural and functional abnormalities in tumor microenvironment. They are irregular in size and shape and lack the conventional hierarchy and the identifiable features of arteries and veins [16, 17]. Figures 4(a)–4(p) display the SHG, TPEF, overlaid SHG/TPEF and corresponding H&E-stained image of the abnormal blood vessels in tumor microenvironment, respectively. The first row in Figure 4 displays the MPM (a, b, and c) and H&E-stained (d) images of a large number of microvessels and macrophages in the tumor microenvironment. The microvessels (yellow arrow) and macrophages (white arrow) were detected by TPEF signals. In tumors, the abnormal blood vessels are of central importance in tumor growth and in cancer detection and treatment procedures, while the density of macrophages is considered as a poor prognostic marker for many cancers, including breast cancer. Tumor-associated macrophages (TAMs) are critical for tumor metastasis [18, 19]. They facilitate angiogenesis by the synthesis of angiogenic factors, regulate collagen fibrillation, and promote tumor cell motility [20]. It can be obviously seen from the MPM images that there are a lot of microvessels around macrophages. Therefore, macrophages are the center of the invasion microenvironment and important drug targets for tumor treatment [21]. For a clearer view of macrophages, Figures 5(a) and 5(b) show an enlarged view of the green rectangular region. The macrophages (white arrow) are large in size, as well as rich and foamy in cytoplasm. Microvessels (yellow arrow) are very clear because there are lots of red blood cells in vessel lumen and these cells can emit strong TPEF signals. Some lymphocytes (blue arrow) are scattered around blood vessels. These cells could be identified because the nucleus is almost filled with whole cells and the cytoplasm is rare, thus producing weaker TPEF signals. Therefore, the morphological features of these blood vessels in tumor microenvironment can be clearly seen from the MPM imaging and can be clearly distinguished from the surrounding cells. The MPM images of the above various components correspond well with the H&E-stained images. These components were confirmed by an experienced pathologist.

In Figures 4(e)–4(h), there are many blood vessels (yellow arrow) around the tumor cells (pink arrow), and the morphology of blood vessels is quite different. Compared with the normal blood vessels in Figure 3, they are irregular in size and shape, often malformed and more tortuous, and have thin walls. Blood vessels in tumors differ from normal vessels in many ways. They have irregular structures and abnormal functions. Irregularly shaped blood vessels may prevent drug delivery to tumor cells, thus promoting tumor progression [16, 17, 22]. From the top of the MPM images, it is clear that tumor cells (pink arrow) tend to migrate to blood vessels along the direction of collagen fibers (white arrow). In the breast tumor, the TPEF imaging can clearly distinguish single tumor cell and depict the distribution of cells. The SHG imaging clearly revealed the linearized collagen bundles in tumor microenvironment. From the enlarged view (Figures 5(c)–5(d)) of the pink rectangles, we can see the morphological features of tumor cells and collagen more clearly.

Figures 4(i)–4(l) depict the microstructure of the abnormal blood vessel around the tumor. The wall of blood vessel is incomplete and lacks the tight endothelial cells necessary for normal barrier function. The endothelial cells of normal blood vessels are relatively intact and act as a selective barrier, strictly controlling the exchange of substances between the blood and the surrounding tissue [23]. This abnormal structure results in increased vascular wall permeability, providing a structural basis for continuous infiltration of plasma and blood cells involved in inflammatory reactions into pathological tissues. As shown in Figures 4(i)–4(l), there are a large number of erythrocytes around the blood vessels. Hemorrhage is a manifestation of the defective vessel wall barrier function in tumors [24]. These endothelial gaps in the tumor blood vessels could also act as channels for tumor cells into the circulation, thus forming metastases [25]. According to these results, the abnormal blood vessels in tumor can be correctly identified by MPM imaging.

Figures 4(m)–4(p) show the blood vessels (yellow arrow) that have been invaded by tumor cells (white arrow) in the tumor microenvironment. This indicates that the tumor cells have metastasized or has the possibility of metastasis. From the SHG image, straight collagen fibers (blue arrow) are aligned parallel to the blood vessel's boundary. On the other side of the vessel, bundles of collagen fibers (red arrow) are perpendicular to the blood vessel's boundary. At the same time, SHG imaging clearly shows that the basement membrane (pink arrow) of blood vessels is destroyed, which makes it easier for tumor cells to invade blood vessels. Tumor cells preferentially invade along straight collagen fibers, thereby promoting intravasation [26]. Compared with the MPM image, the collagen fibers in the H&E-stained image cannot be clearly recognized. This ability to identify collagen fibers is an advantage of MPM imaging.

**Figure 6:**
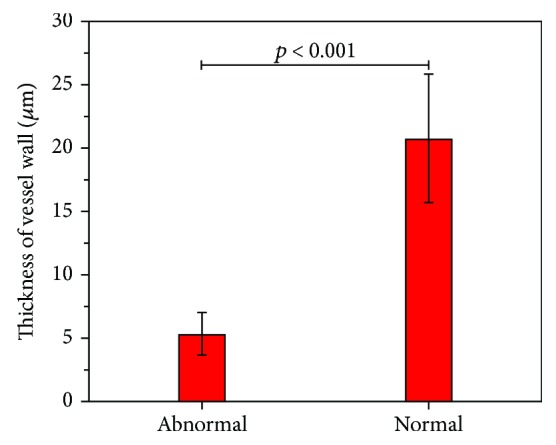
Thickness of the normal and abnormal blood vessel walls. Error bars indicate standard deviation.

To quantify the difference in morphological features between normal and abnormal blood vessel, the thicknesses of the normal and abnormal blood vessel wall were calculated.  Figure 6 displayed the quantitatively analyzed results of normal and abnormal vascular walls. The wall of the abnormal blood vessel was 5.38 ± 1.69 *μ*m (*n* = 60), while that of the normal blood vessel was 20.81 ± 5.09 *μ*m (*n* = 60), because tumor vessels have poor vascular stability, poor quality, and thin walls [25]. The differences between the two groups are also highly significant (*p* < 0.001). The statistical analysis was performed using the IBM SPSS Statistics 24.

## 4. Discussion

MPM imaging has the potential to be applied to intraoperative label-free pathological diagnosis [27]. It is more and more widely used in medical imaging including blood vessel imaging. In this study, there were several endogenous fluorophores such as collagen, elastin fibers, endothelial cells, and smooth muscle of the blood vessel wall. The morphological details of endogenous fluorophores of the blood vessel wall were clearly shown in the MPM images. MPM can clearly display the layer structure of the vascular wall. Then, the emission spectra of the different layers of the blood vessel wall were acquired. We can distinguish the three-layer structure of the vascular wall from the spectral information and correctly identify different types of blood vessels via MPM imaging. It can be seen that MPM had the ability to visualize various features of blood vessels in tumor microenvironment, and this may provide assistant diagnostic indicators for pathologists and surgeons. Compared with traditional H&E staining, MPM was more effective in distinguishing collagen fibers.

It is now well known that interaction between tumor cells and their microenvironment is important for tumor progression and metastasis [28]. In tumor microenvironment, macrophages are important promoters of angiogenesis and the interaction between macrophages and tumor cells results in invasion and egress of tumor cells into the blood vessels [20]. Similarly, alignment of collagen fibers directs tumor cell intravasation [26]. High vessel permeability may promote metastasis and spread of cancer. There is universal agreement that vascular networks in tumors play a central role in the growth, diagnosis, and treatment of tumors. Without blood vessels, tumor cannot grow beyond a critical size or metastasize to other organs. Similarly, without blood transport, we might not be able to deliver anticancer drugs to tumor areas effectively [29]. Thus, advanced imaging techniques are needed to monitor vascular changes in patients. Various imaging modalities may be used for vascular imaging, such as MRI, CT, PET, and fluorescence imaging. However, these imaging technologies still have some disadvantages, including low resolution, poor sensitivity, and requiring contrast medium and exogenous fluorescence markers. The advantage of MPM is that it can visualize blood vessels at cellular and molecular levels without using these external markers. In this study, MPM imaging can accurately identify the microstructure of blood vessels and clearly observe the changes of blood vessels in tumor microenvironment.

## 5. Conclusion

In conclusion, we demonstrated that label-free MPM is a promising technique for studying the morphometric properties of the blood vessel wall. In recent years, the development of two-photon microendoscopic imaging technology provides the possibility for in vivo brain imaging [30]. If multiphoton endoscopy technology is standardized, it will allow real-time noninvasive imaging of the human body, thus avoiding unnecessary biopsy. With miniaturization of MPM endoscopy, MPM imaging will provide accurate intraoperative diagnosis of the vascular morphology in vivo.

## Data Availability

The data used to support the findings of this study are available from the corresponding author upon request.
